# Photonic Nanojet‐Mediated Optogenetics

**DOI:** 10.1002/advs.202104140

**Published:** 2022-02-20

**Authors:** Jinghui Guo, Yong Wu, Zhiyong Gong, Xixi Chen, Fei Cao, Shashwati Kala, Zhihai Qiu, Xinyi Zhao, Jun‐jiang Chen, Dongming He, Taiheng Chen, Rui Zeng, Jiejun Zhu, Kin Fung Wong, Suresh Murugappan, Ting Zhu, Quanxiang Xian, Xuandi Hou, Ye Chun Ruan, Baojun Li, Yu Chao Li, Yao Zhang, Lei Sun

**Affiliations:** ^1^ Department of Physiology School of Medicine Jinan University Guangzhou 510632 China; ^2^ Department of Biomedical Engineering The Hong Kong Polytechnic University Hong Kong SAR 999077 China; ^3^ Institute of Nanophotonics Jinan University Guangzhou 511443 China

**Keywords:** light focusing, microspheres, optogenetics, photonic nanojets

## Abstract

Optogenetics has become a widely used technique in neuroscience research, capable of controlling neuronal activity with high spatiotemporal precision and cell‐type specificity. Expressing exogenous opsins in the selected cells can induce neuronal activation upon light irradiation, and the activation depends on the power of incident light. However, high optical power can also lead to off‐target neuronal activation or even cell damage. Limiting the incident power, but enhancing power distribution to the targeted neurons, can improve optogenetic efficiency and reduce off‐target effects. Here, the use of optical lenses made of polystyrene microspheres is demonstrated to achieve effective focusing of the incident light of relatively low power to neighboring neurons via photonic jets. The presence of microspheres significantly localizes and enhances the power density to the target neurons both in vitro and ex vivo, resulting in increased inward current and evoked action potentials. In vivo results show optogenetic stimulation with microspheres that can evoke significantly more motor behavior and neuronal activation at lowered power density. In all, a proof‐of‐concept of a strategy is demonstrated to increase the efficacy of optogenetic neuromodulation using pulses of reduced optical power.

## Graphical abstract

Activation of ChR2 by blue light can be enhanced by polystyrene (PS) microsphere in vitro. PS microsphere facilitates the regulation of neuron activity by opening of ChR2 ex vivo. Photonic nanojet‐mediated optogenetics by PS microsphere improves the regulation of targeting neuronal activity in vivo.

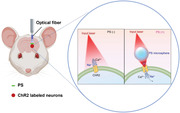

## 1 Introduction

Optogenetics has been widely recognized as perhaps the most powerful tool for neuromodulation in the past decade, providing a significant boost to neuroscience research.^[^ ^1^ ^]^ Allowing for millisecond temporal resolution in regulating neuronal activity and potential single‐cell spatial resolution, optogenetics could not only further our understanding of brain function but also reveal possible therapies for neuropsychiatric disease.^[^ ^2^, ^3^, ^4^ ^]^ Neuronal activity modulated by optogenetics mainly depends on optical gating of opsin channels that have been exogenously expressed in specific types of neurons. The opening of opsin channels by milliseconds‐long optical stimulation enables the generation of action potential or neuronal inhibition, making optogenetics a versatile and adaptable neuromodulation approach. However, there remains an important issue that opsin nonexpressing cells are often activated at high optical intensity through other mechanisms (e.g., photothermal effects),^[^ ^5^ ^]^ although only opsin‐expressing cells are expected to be the target. Therefore, the accuracy and specificity of optogenetics are not only a result of genetic modification of opsins, but also good control of optical power density.

The delivery of light in optogenetics often requires the implantation of an optical fiber located within 100 µm of the target neuron populations. Optical attenuation by tissue scattering and absorption, at the most commonly utilized wavelengths of 400–600 nm, causes an exponential decay of power density in brain tissue and results in quick power decrease. Power drops by 50% at a depth of 100 µm and 90% at 200 µm have been reported,^[^ ^6^, ^7^ ^]^ thus limits the possible stimulation area only close to the tip of the optical fiber with sufficient photons.^[^ ^8^, ^9^ ^]^ This complicates the balance between specificity and efficacy, because a higher optical power density is often applied to compensate for the power loss, which could lead to nontargeted cells being affected. Therefore, a strategy that could enable effective stimulation of neurons with lower optical power density could be highly desirable for the optogenetic paradigm.

A photonic nanojet (PNJ) is a highly focused light beam generated close to the shallow side of an illuminated lossless dielectric microcylinder or microsphere.^[^ ^10^, ^11^, ^12^ ^]^ It features subwavelength focal region and has been proved to have broad applications in single‐molecule detection,^[^ ^13^ ^]^ optical trapping,^[^ ^14^, ^15^ ^]^ super‐resolution imaging,^[^ ^16^, ^17^ ^]^ fluorescence enhancement,^[^ ^18^, ^19^ ^]^ etc. In the present study, we harnessed the PNJ effect of transparent polystyrene microspheres (PS) as microlenses to focus the incident light and significantly enhance the optical power density to the neighboring neurons to overcome the strong optical attenuation problem in brain tissue. In vitro results showed that PNJs generated by PS microspheres boosted the inward currents of target cells by nearly 132% and significantly reduced the required power density threshold to evoke action potentials, compared to cells without PS. PS‐mediated optogenetic stimulation also enabled evocation of motor behavior and neuronal activation of freely moving mice at lower power density. Thus, we provide proof‐of‐concept for a straightforward and practical method of enabling efficient optogenetic stimulation with light at lower power density with minimal off‐target effect.

## 2 Results

### 2.1 Optogenetics Enhancement Scheme and Light Focusing by PS Microspheres

**Figure 1:**
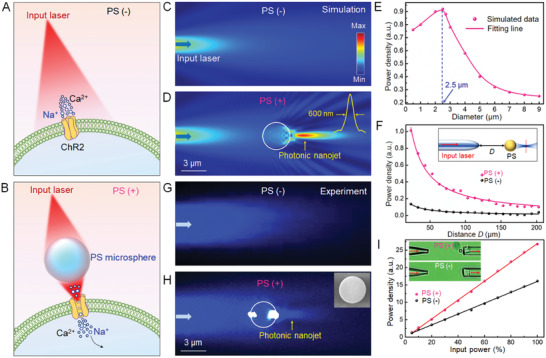
Dielectric microsphere enhanced optogenetics. A) Schematic illustration of optogenetics without a microsphere. The diverging laser beam is ineffective opening the ChR2 channel. B) When a PS microsphere is placed near the ChR2 protein, the beam is focused by the PS microsphere and can successfully open the channel. C) Simulated optical field distribution showing the input light diverging and attenuating along the propagation direction. D) The input light is focused into a small region at the shadowed surface of the microsphere. Inset shows that the waist radius of the focal spot is 600 nm. E) Power density of the focal spot of the PS microsphere as the function of its diameter. F) Power density collected by an optical fiber as a function of the distance between the fiber tip and microsphere. PS(+): pink line, PS(−): black line. Inset shows a schematic of the optical setup for the power measurement. G) Experimental optical field distribution indicates the input light diverges without the microsphere. H) When the microsphere is placed in the optical axis, the input light is focused because the microsphere acts as a microlens. Inset shows a SEM image of the PS microsphere. I) Power density collected by an optical fiber as a function of the input power. PS(+): red line; PS(−): black line. Inset shows the optical setup for the power measurement.

In this study, we used a transparent PS microsphere in vitro and in vivo to focus optical power locally. Our basic idea was that without a PS microsphere (PS(−)), the diverging light beam is ineffective in opening the ChR2 ion channel (**Figure** **1**A). When a PS microsphere (PS(+)) is placed near the cell membrane, the light beam is strongly focused by the microsphere, and the increase in power density is enough to open the channel, allowing cations into the neuron, leading to activation (Figure 1B). A simulation was performed to investigate the light focusing property of PS microspheres using Comsol Multiphysics. In the simulation, the incident light was set as a Gaussian beam at a wavelength of 488 nm launched from a tapered fiber and the refractive index of PS microsphere was set as 1.6. Simulated optical field distribution shows the incident light diverging and attenuating along the propagation direction in the condition of PS(−) (Figure 1C). By placing a PS microsphere at the optical axis, the incident light was focused into a PNJ with a waist radius of 600 nm (Figure 1D). The focusing performance of the PNJ was related to the sizes of PS microspheres. For the 488 nm incident laser beam, the highest power density of the PNJ occurred at a microsphere diameter of 2.5 µm (Figure 1E). The calculated enhancement factor, which was defined as the power density of PS(+)/PS(−), decreased with the increase of the distance between the input fiber and microsphere (Figure 1F). The enhancement factor was calculated as 2–5 when the distance was less than 200 µm. Therefore, the distance between the fiber and microsphere was controlled within 200 µm for subsequent optogenetic experiments. The focusing property of PS microspheres was then experimentally investigated via Tyndall effect^[^ ^21^ ^]^ of nanoparticles suspended in the PS solution (Figure 1G,H). The PS microspheres were found capable of generating PNJs with the highest power density at microsphere diameter of 3.0 µm. The experimental results indicated a slight deviation with the simulation (2.5 µm) because the numerical model of the PS microsphere was totally homogeneous and transparent in the simulation. The enhancement factor was measured as ≈2 at a 150 µm distance between the fiber and microsphere with different incident optical powers (Figure 1I). Based on these data, we chose PS microspheres with a diameter of 3.0 µm for converging light and optical stimulation in subsequent in vitro and in vivo experiments.

### 2.2 Enhancement in Activation of ChR2 by PS Microspheres In Vitro

**Figure 2:**
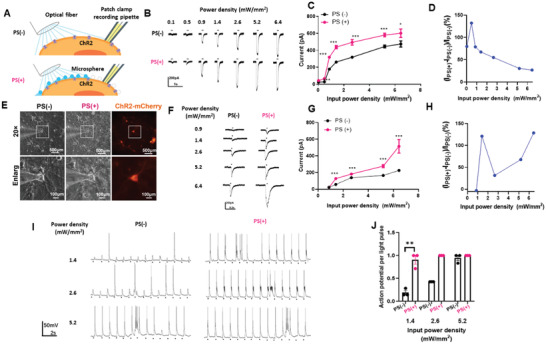
PS microsphere enhances light‐evoked neuron activity via ChR2. A) Schematic configuration for in vitro experiment. Whole‐cell patch clamp recording was conducted in ChR2‐expressed cells stimulated by blue light in the presence of PS (PS(+)) or not (PS(−)). B) Inward currents induced by a 100 ms light pulse with or without PS in 293T cell with ChR2. C) The curve between input power density and inward currents induced by light with PS or without PS in 293T cells, *n* = 5, ^*^ *p* < 0.05, ^**^ *p* < 0.01, ^***^ *p* < 0.001, unpaired two‐tailed *t*‐test. D) Inward currents of the target neurons as a function of the input power density. E) Primary hippocampal neurons expressing ChR2‐mCherry in bright filed and 488 nm excitation without PS or with PS (scale bar 100 µm). F) Inward currents induced by 20 ms light pulse with or without PS in primary hippocampus neuron with ChR2. *n* = 10, ^***^ *p* < 0.001 unpaired two‐tailed *t*‐test. G) The curve between input power density and inward currents induced by light with PS or without PS in primary hippocampus neuron. H) Inward currents of the target neurons as a function of the input power density. I) Current clamp recording of hippocampus neuron stimulated with 20 ms pulse light without or with PS. J) Success rate of generation of action potential evoked by light pulse in different power density. *n* = 3, ^**^ *p* < 0.01, unpaired two‐tailed *t*‐test.

The ability to enhance neural activation by the PS microsphere‐generated PNJs was first investigated in cells in vitro. Broadly, ChR2‐expressing cells were stimulated with 488 nm light with or without PS present, and whole cell patch clamp recording was used to record both channel and neuronal activity (**Figure** **2**A). To show the effect of the PNJs in opsin activation, ChR2 was overexpressed in 293T cells with lipofectamine‐mediated plasmid transfection, in which very few ion channels are expressed endogenously.^[^ ^20^ ^]^ With pulsed light stimulation (wavelength: 488 nm; pulse duration: 100 ms), ChR2‐293T cells showed inward current intensities dependent on the optical power density (Figure 2B), indicating the successful expression of ChR2. However, in the presence of microspheres (PS(+)), inward current intensity was significantly increased compared with that in the absence of the microspheres (PS(−)) at the same optical power density (Figure 2B,C). The enhancement by PS ascended to a maximum of ≈150% at an optical power density of 0.5 mW mm^−2^ and decreased at higher densities due to the saturation of ChR2 channel opening^[^ ^21^ ^]^ (Figure 2D). In addition, to prove our concept that PS works as microlenses but did not depend on its own material characteristics, TiO_2_ was applied to investigate its effect of enhancing the activation of the ChR2‐293T cells, and the results showed the similar effects with that of PS microspheres (Figure S1, Supporting Information). We next investigated whether the microspheres could enhance the activation of primary neurons expressing ChR2. Primary cultured hippocampal neurons were transduced by AAV9‐hSyn‐ChR2‐mCherry, and channel expression was verified by neurons showing red fluorescence (Figure 2E) Notably, PS microspheres are visible near the neurons using brightfield microscopy. Pulsed optical stimulation (pulse duration: 20 ms) induced power‐density‐dependent inward currents, and PS(+) neurons showed significantly high inward current intensity at all tested power densities (Figure 2F,G). Interestingly, the enhancement by PS reached a peak of ≈121% at 1.4 mW mm^−2^ and then dropped between 1.4 and 2.6 mW mm^−2^, but ascended further at higher power densities (>2.6 mW mm^−2^), which is not consistent with the case of ChR2‐293T cells (Figure 2H). This is because neurons express other voltage‐dependent cation channels which contribute to inward currents following by the activation of ChR2 at high power.^[^ ^22^ ^]^ Current clamp recording revealed that the rate of generated action potentials in primary neurons (power density: 2.0 mW mm^−2^; pulse duration: 10 ms) was significantly greater in PS(+) cells at lower power densities than PS(−) cells (Figure 2I,J). PS(+) and PS(−) showed a similar action potential rate at 3.1 mW mm^−2^, suggesting that ChR2 opening was saturated at this density (Figure 2J).

### 2.3 Regulating Neuron Activity with PS Microspheres Ex Vivo

**Figure 3:**
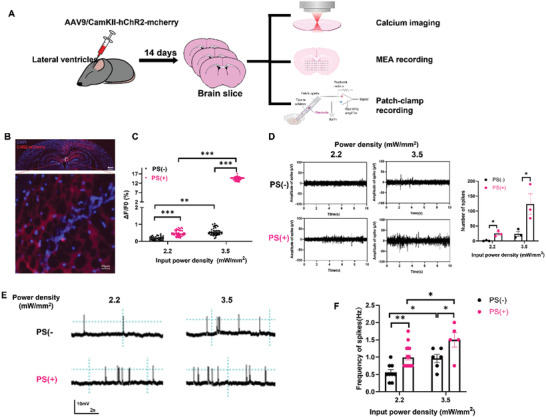
Microsphere‐based optogenetics in modulating neuron activity in brain slice. A) Schematic illustration of ex vivo experiments for testing microsphere‐based optogenetics. Newborn mice were injected in lateral ventricle with AAV9/CamKII‐hChR2‐mCherry, and brain slice were collected for calcium imaging, MEA recording as well as membrane potential patch‐clamp recording after 14 days virus injection. B) Representative images of ChR2‐mCherry expression in the brain after 14 days virus infection. (Scale bar in large field image: 100 µm; scale bar in enlarged image: 25 µm.) C) Bar chart shows the mean ± SEM of fifty neurons’ Ca^2+^ fluorescence changes induced by light stimulation. *n* = 50, ^**^ *p* < 0.01, ^***^ *p* < 0.001m, unpaired two‐tailed *t*‐test. D) Representative traces of light‐induced neuronal activity in brain slice with ChR2 in with or without PS, obtained by MEA recording as well bar chart of the mean ± SEM that show the firing number, *n* = 3, ^*^ *p* < 0.05 unpaired two‐tailed *t*‐test. E) Membrane potential recording e after 1 min light stimulation in the brain slice with ChR2 in with or without PS. F) Summary bar graph showing the frequency of neuronal firing after 1 min of light stimulation with or without PS. *n* = 6–9, ^*^ *p* < 0.05, ^**^ *p* < 0.01, unpaired two‐tailed *t*‐test.

We next evaluated the enhancement effect of our proposed strategy ex vivo. In order to exclude the effects of inhibitory neuron, AAVs coding for CamKII‐ChR2‐mCherry but not hsyn promoter used in the in vitro experiment were injected into the lateral ventricles of neonatal mice. The mice were sacrificed 14 days later with their brains were recovered and sliced for calcium imaging, microelectrode array (MEA) recording as well as membrane potential recording with patch clamp (**Figure** **3**A). ChR2 was demonstrated to be successfully expressed in the hippocampus and partially cortex (Figure 3B). X‐Rhod‐1 was used as the intracellular calcium indicator in living brain slices. Fifty neurons expressing mCherry were selected, and the changes of their intracellular calcium stimulated with blue light for 1 min were recorded (Figure 3C). Optogenetic stimulation at 2.2 mW mm^−2^ could not increase the intracellular calcium in most neurons, even in PS(+) cells, but 3.5 mW mm^−2^ stimulation was significantly enhanced in PS(+) by ≈30% (Figure 3B). MEA recording was also performed to detect extracellular electric signals in brain tissue. The PS(+) showed significantly more spikes after both tested power densities, whereas PS(−) only showed a few spikes at 3.5 mW mm^−2^ (Figure 3D). Moreover, membrane potential recording with patch clamp was also applied to investigate the effect of PS on the neural firing after blue light stimulation. The results showed that neurons in the presence of PS exhibited more spikes than without PS after 1 min blue light stimulation (Figure 3E,F). These results were consistent with those from calcium imaging and MEA experiments. Thus, ex vivo experiments also confirmed the ability of PS microspheres to significantly enhance the efficiency of optogenetic stimulation at the same power density.

### 2.4 Enhanced Optogenetics by PS Microspheres In Vivo

**Figure 4:**
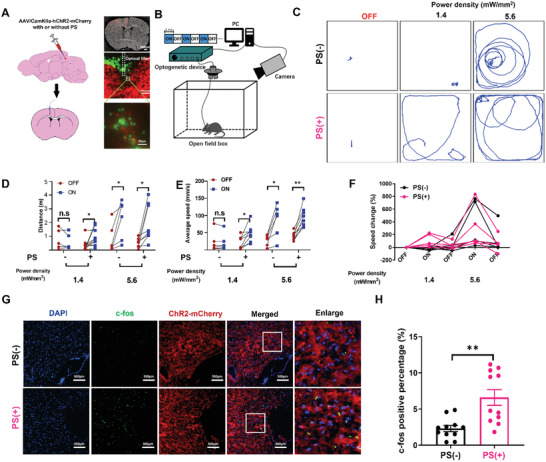
Activation of M1 neurons by microsphere‐based optogenetics induces locomotion A) Experimental scheme of the labeling approach to express either ChR2‐mCherry with or without PS microsphere into the M1 area. Virus with PS or without PS was injected into M1 region. PS with green fluorescent was applied to visualize the location of PS and optical fiber. B) Illustration of the light‐stimulation setup used in the optogenetic sessions. Optogenetic testing consisted of 1 min epoch with alternating light manipulation (OFF–ON–OFF–ON–OFF). Light stimulation (465 nm, 10 ms square pulses at 20 Hz. C) Representative trace recordings of mice with light OFF or ON at varying power density. D) Distance traveled during the light‐OFF or ON periods at varying power density. *n* = 5, ^**^ *p* < 0.01, unpaired two‐tailed *t*‐test. E) Average speed in light‐OFF and ON period with light at varying power density. *n* = 5, ^**^ *p* < 0.01, unpaired two‐tailed *t*‐test. F) Changes of speed ((OFF–ON (1.4 mW mm^−2^)–OFF–ON (5.6 mW mm^−2^)–OFF). G) Representative images of M1 neurons expressing c‐Fos after light stimulation (1.4 mW mm^−2^)1 of ChR2 with or without PS (scale bar 100 µm). H) Nuclear c‐fos percentage per slice imaged in the M1 in with or without PS. Bar chart represents means ± SEM the percentage of c‐Fos^+^ cells of per imaged slice. *n* = 10, ^**^ *p* < 0.01, unpaired two‐tailed *t*‐test.

As a further test of the system's efficacy, we tried to apply our PNJ‐enhanced optogenetics scheme in vivo. The optical neural interface developed by the Deisserroth et al. is widely used for optogenetic neuromodulation^[^ ^23^ ^]^ but blue light used to gate ChR2 undergoes significant scattering and absorption in tissue leading to attenuation of power density. To demonstrate that the PS microsphere‐based optical neural interface utilization of photons for neuromodulation in vivo, we used a locomotion‐related behavior assay.^[^ ^24^, ^25^ ^]^ AAVs coding for CamKII‐ChR2‐mCherry, with or without PS microspheres, were injected into M1 region, which controls motor activity (**Figure** **4**A). Optical fibers were implanted in the M1 regions (depth: 50 µm) of a C57BL/6J mice 21 days post‐transduction. To confirm the spatial distribution of the PS microspheres, some mice were injected with green fluorescent‐labeled PS along with the virus. After implantation of optical fibers, we observed that ChR2‐mCherry‐labeled neurons were surrounded by PS with green fluorescence, below the optical fibers, representing the location of the microspheres (Figure 4A). An open‐field behavior test was conducted during which blue light was switched from 1 min ON to 1 min OFF, and the motion trail of the mice was recorded (Figure 4B). At a relatively low optical power density (1.4 mW mm^−2^), PS(−) mice showed the same activity as when the light was off, but the PS(+) mice obviously increased their locomotor behavior (Figure 4C) Notably, when the power density was increased to 5.6 mW mm^−2^ both PS(−) and PS(+) obviously increased locomotion, indicating the successful gating of ChR2 in both. PS(+) mice covered significantly increased distances and significantly increased their speed when the 1.4 mW mm^−2^ light was switched on at (Figure 4D–F). Both groups showed significant increases in locomotion at 5.6 mW mm^−2^. The M1 regions of mice treated with 1.4 mW mm^−2^ light were later stained for the activation marker c‐Fos. PS(+) mice showed significantly greater c‐Fos expression in the region, indicating effective enhancement of neuronal activation consistent with the locomotion data (Figure 4G,H). Crucially, we observed ChR2 saturation at higher power density (5.6 mW mm^−2^), such that there was no significant difference between the PS(−) and PS(+) groups. This confirms that the ChR2 channels were successfully encoded and were functioning as expected These results show that the optical power threshold for gating ChR2 in vivo was considerably reduced (≈75% reduction) by the effect of PNJs, confirming that the optogenetic stimulation was indeed enhanced by the presence of PS microspheres.

### 2.5 Discussion and Conclusions

This study shows the application of PS microspheres as the microlenses to enhance the localized power density and reduce the optical power threshold for enhanced optogenetics with better efficacy and less off‐target effect through in vitro, ex vivo, and in vivo experiments. These experiments demonstrate that PS microspheres can significantly enhance cellular calcium dynamics, inward current, electrical activity and signaling, and motor behavior in animals. Crucially, the PNJ effect of the PS microspheres greatly boosted the activation of neurons in vivo, helping to circumvent the issue of optical attenuation and scattering in brain tissues. The proposed PNJ‐enhanced optogenetics could enable even greater spatial accuracy in optogenetic neuromodulation due to the reduction of off‐target effects and the concentration of optical power around the microspheres. This research provides the first documentation of the microsphere‐aided optogenetic enhancement.

Although optogenetics has become a powerful technique in facilitating the study of physiological functions of specific brain regions and even single cells, researchers always have concerns that nontargeted neurons could be inhibited by high‐intensity optical stimulation, which may lead to false interpretation of the phenotypes by optogenetics not only because of the targeted neurons but also off‐targeted neurons due to the photonic effect. Much effort has been made to develop new opsins, activatable by longer wavelength light to avoid thermal and photonic toxicity. For example, redshifted opsin channels have been applied in optogenetics, enabling lower‐energy photons to regulate neuron activity directly compared with those at shorter wavelengths.^[^ ^26^, ^27^ ^]^ In parallel, improving the photonic distribution is another possible strategy. Our study used microspheres as lenses to generate PNJs that concentrate the optical intensity in a localized area, thereby allowing light of lower power density to successfully stimulate targeted cells. That said, we consider the PS microspheres representative of the enhancement that may be achieved by microsphere lensing in general, not specifically microspheres of this material. Indeed, being made of a foreign material may lead to some undesirable biocompatibility issues, especially in the brain. Notably, PS microsphere has been shown biocompatible with the brain tissue.^[^ ^28^ ^]^ In a previous study by the authors of this paper, we have demonstrated that endogenous microstructures (e.g., red blood cells) existing in the body can also be applied as microlenses.^[^ ^29^ ^]^ Since PS microspheres were used in this work to generate PNJs, our study can be viewed as a proof‐of‐concept example for the microlens‐based enhancement strategy for optogenetics. In spite that microlens‐based optogenetics enabled precise regulation of targeted neuronal activity, we have to confess the requirement of invasive optical fiber implantation still cannot be avoided. Therefore, other strategies for light delivery, such as focused ultrasound to guide the propagation and emission of light in the local brain tissue noninvasively can become complementary to the nanoject based optogenetics in the future studies.^[^ ^30^, ^31^ ^]^

Using microspheres for the formation of PNJs offer the possibility of tailoring their physical properties depending upon the intended application. Opsins that have been applied in optogenetics can be activated by light at different wavelengths. For example, ChR2, the most popular excitatory opsin, is gated by wavelengths from 460 to 490 nm, while NpHR, a popular inhibitory opsin, is sensitive to yellow light at 560 nm. Given the diversity of wavelengths required to stimulate commonly used opsins, the refractive index and size of the microspheres can be optimized for better performance. For example, a longer wavelength generally results in a larger focal length at the same microsphere size. Thus, when a longer wavelength is required for stimulation, a higher refractive material can be used to maintain the size and the depth of the focus, and vice‐versa, in which TiO2 (a higher refractive index (*n*_o_ = 2.73, *n*_e_ = 3.06)) may be the better choice that works as the microlenses. We consider this study as a guide for the future development and applications of microsphere‐enhanced optogenetics.

To conclude, we have applied PS microspheres as the microlenses to enhance the localized field intensity and reduce the optical power threshold for optogenetics. The PNJ effect of the PS microspheres greatly boosted the inward currents of the target neuron at a relatively low optical power density, circumventing the optical attenuation and scattering issue in brain tissues. The proposed PNJ‐enhanced optogenetics, which enables a more spatially refined modulating of the neuron activity and avoids the side effects on nontarget neurons, may provide new light and thought in the development of the highly precise optogenetics.

## 3 Experimental Section

### Preparation of PS Microsphere Solution

PS microspheres (Huge Biotechnology Co., Ltd, Shanghai, China) with an average diameter of 3 µm and a refractive index of 1.6 were used in the experiments. To improve the biocompatibility, the surfaces of the PS microspheres were modified with amino acids. PS microsphere solution was diluted to a suitable concentration of ≈5.0 × 10^3^ µL^−1^ with PBS buffer solution. The solution was then treated with an ultrasonic vibrator to prevent the aggregation of microspheres. Finally, the microspheres were injected into cell suspension or tissues with a microinjection syringe.

### Animals

Eight‐week‐old C57BL/6J mice were used for ex vivo and in vivo experiments. Standard housing conditions, with 12 h light/dark alternation and food and water available ad libitum, were provided for all animals. All animal experiments were conducted in accordance with the university on animal experimentation, and approval by the Animal Ethics Committee and The Hong Kong Polytechnic University was obtained for all related procedures.

### Cell Culture

All cells were incubated in a humidified incubator maintained at 37 °C with 5% CO2. Primary neurons were obtained surgically from mouse embryos at day 16 as described previously.^[^ ^33^ ^]^ Cortices were first immersed and sectioned in ice‐cold Neurobasal medium and then incubated for 15 min in 0.25% trypsin–EDTA in a water bath at 37 °C. Cells were then collected by centrifuging and washing with Neurobasal medium, mechanically triturated with a pipette and then allowed to settle. The supernatant was discarded, cells were resuspended in medium containing 10% FBS + 2% B27 serum‐free supplement + 0.25% l‐glutamine, and seeded in 35 mm dishes containing poly‐l‐lysine (PPL)‐coated coverslips at 5 × 10^5^ cells per dish. The medium was changed after 24 h to Neurobasal + 0.25% l‐glutamine + 2% B27 +1% penicillin–streptomycin and half rechanged every 72 h.

The HEK‐293T cell line were purchased from ATCC. Cells were cultured in DMEM + 10% FBS + 1% penicillin–streptomycin.

### Viral Transduction

High‐titer viruses (pAAV‐CaMKIIa‐hChR2(H134R‐mCherry)) and (pAAV‐hsyn‐hChR2(H134R‐mCherry)) were obtained commercially and stored at −80 °C prior to use. The ChR2‐H134R gene was fused with that of the fluorescent reporter mCherry. The CaMKII*α* promoter was used to preferentially transduce only excitatory neurons. Virus diluted 1:100 in PBS was added to primary neuron cultures at DIV7 at room temperature. For each dish with 5 × 10^5^ cell density, 10^10^ genome copies were directly added into the cell medium. The plates were gently shaken and placed in the incubator for 3–5 days and the cell condition and fluorescence were closely monitored. Plates showing cells with successful transduction (evaluated by bright fluorescence) were used in further experiments.

### Patch Clamp

Whole cell patch clamp was used for recording the inward currents and membrane potential in vitro. Borosilicate glass patch pipettes (Vitrex, Modulohm A/S, Herlev) were pulled by using a micropipette puller (P‐97, Sutter Instrument Co.) and filled with pipette solution to have a resistance of 5–7 MΩ. Current clamp was applied for measuring membrane potential and voltage clamp was applied for measuring inward current. Transduction was confirmed by fluorescence imaging. The cells were put in bath solution containing (mM): NaCl 130, KCl 5, MgCl_2_ 1, CaCl_2_ 2.5, Hepes 20, and glucose 10 (pH 7.4). The pipettes filling solution contained 1 × 10^−3^ m MgCl_2_, 138 × 10^−3^ m KCl, 10 × 10^−3^ m HEPES, 10 × 10^−3^ m NaCl, and d‐mannitol compensated for osm 290 (pH 7.2). Digidata 1440B (Axon Instruments) and amplifier (Axopatch‐700A, Axon Instruments) together with pClamp Version 9 software were used for data recording. The data were analyzed by using Clampfit 10.0. Neuronal membrane potential in acute brain slice recording was conducted with EPC10 patch clamp amplifier (HEKA) equipped with upright microscope with fluorescence (Olympus BX51W1). The pipette solution was the same with cultured cells and the live brain slice was bathed with gas‐bubbled artificial cerebral spinal fluid (ACSF) containing (mM): NaCl 124, Glucose 10, KCl 3, NaHCO_3_ 26, CaCl_2_ 2, NaH_2_PO_4_ 1.25, and MgSO_4_ 2. The gas comprised of 95% O_2_ and 5% CO_2_ to maintain a pH of 7.4 in ACSF.

### Intracerebroventricular Viral Injection

Viral injection to the intracerebroventricular region of the neonatal mouse brain was conducted for brain‐wide transduction as described in detail previously.^[^ ^34^ ^]^ Briefly, P0 pups were anesthetized on ice and then virus was injected using a 10 µL syringe with a 32 G needle within 6 h of their birth. The pups’ heads were gently wiped with a cotton swab soaked in 70% ethanol before injection. 3/5 of the distance from the eye to the lambda suture was identified as the injection point. Then 1 µL thawed virus aliquot was loaded into the injection syringe and the pup was laid on its side, with its head directly under the syringe. The syringe needle was placed perpendicular to the skull and inserted to a depth of 3 mm. Both injection and withdrawal of the needle were done slowly. Pups were then labeled and placed near a heat source to allow recovery of body temperature and returned to their mother's cage. The condition of the pups were monitored every day in the first few days after injection.

### Optical Stimulation Methodology

For in vitro and ex vivo experiments, blue light pulses were generated using optogenetic manipulator (Inper, China). Optical fiber with 200 µm diameter was applied for light deliver, yielding a blue light intensity from 0.1 to 6.5 mW mm^−2^, measured with a power meter (1815‐C; Newport). The diameter of light spot was adjusted with the aid of microscope according to altering the distance from the tip of optical fiber to the cells. The light pulse lasted 100 ms in 293T cells and 20 ms in primary neuron, respectively.

### Acute brain slice preparation and calcium imaging

Two weeks after intracerebroventricular viral injection, mice were anesthetized by isoflurane 5% in a carbogen gas mixture delivered to an anesthesia induction chamber at 2 L min^−1^. No reflexes in response to foot and paws pinch was observed and then the mice were decapitated. Brains were taken out quickly and sliced in ice‐cold and gas‐bubbled artificial cerebral spinal fluid (ACSF) containing (mM): NaCl 124, Glucose 10, KCl 3, NaHCO_3_ 26, CaCl_2_ 2, NaH_2_PO_4_ 1.25, and MgSO_4_ 2. The gas comprised of 95% O_2_ and 5% CO_2_ to maintain a pH of 7.4 in ACSF. Coronal sections 300 µm thick were sliced by using a vibrating microtome (Leica VT1200S) and recovered in 37 °C gas bubbled ACSF for 1 h before experiments. The prepared acute brain slices were stained with a fluorescent calcium indicator X‐Rhod‐1, AM (X‐14210, Invitrogen) at 4 × 10^−6^ m final concentration in the rACSF with gas bubbling at room temperature. A stained slice was then placed into a confocal dish for microscopy.

The stimulation and imaging system consists of an inverted fluorescence microscope (IX73, Olympus) with Cy3 filter and a sCMOS camera ((ORCA‐ Flash4.0 LT Plus C114400‐42U30, Hamamatsu)) and 488 nm light shed from top down. Slices were imaged for 2 min before stimulation for baseline calculation, then stimulated with only one shot (10 ms) blue pulse and imaged for another 5 min. The stimulation period (10 ms) was not imaged. During imaging, a perfusion pump continuously pumped fresh bathing solution (gas‐bubbled rACSF) to keep the brain slice in good condition.

### MEA Recording

Multielectrode array (MEA) recording was performed by USB‐MEA256‐System, which included an amplifier, four‐channel stimulus generator and two temperature‐control units maintaining solution and base plate at 33 °C. The MEA chamber was sterilized by UV for 2 h. Mouse brain slices were presoaked in artificial cerebrospinal fluid (ACSF) and centered on the MEA perfused with 3 mL min^−1^ flowing ACSF. Slices were stabilized for 1 h at 33 °C before recording. After stabilization, slices were transferred onto the MEA chamber.^[^ ^32^ ^]^ Fluorescent microscope was used to determine which channels the regions with mCherry expressed were located. The channels with target regions of data were sampled at 25 kHz. Neuronal firing with larger than 50 µV was defined as the significant event in the data analysis.

### Stereotaxic Injection and Fiber Insertion

C57BL/6 mice were anesthetized with xylazine (10 mg kg^−1^) and ketamine (100 mg kg^−1^) in PBS. Hairs above the operation location were shaved and a portion of scalp was excised allowing exposure of the skull. Mice were then fixed onto the stereotaxic apparatus and a hole was drilled on the skull with coordinates AP 0.25 mm, ML −1.5 mm, DV −1.0 mm for M1. Mixture of 500 nL pAAV‐CaMKIIa‐hChR2(H134R‐mCherry virus aliquots and 500 nL microsphere suspension was injected at 0.1 µL min^−1^ speed, followed by holding the injection pipette for 10 min at the injection site. The pipette was then withdrawn slowly with another 5 min pause halfway. The surgery site was disinfected and sutured and mice were returned to their cages. Three weeks later, the mice were anesthetized and underwent surgery again to insert fiber ceramic ferrule at the same coordinates. The fiber was fixed with dental cement pulled by small screws on the skull. Open‐field behavioral recordings were conducted one week after fiber insertion.

### Open Field Behavior Testing

The recording of speed, mobile time, mobile episodes, and distance traveled of mice was conducted within a 40 cm × 40 cm × 50 cm field box using one video camera. 10 min habituation were allowed to reduce the acclimation noise. Illumination was powered by a blue LED light source (Inper, China). The power density is adjusted from 1.4 to 5.6 mW mm^−2^. Light stimulation protocols were generated using the Inper software with 10 ms pulse width at 20 Hz of light delivery. Mobile time and episodes were defined as periods (excluding rearing and sniffing) when the movement of the mouse center‐point was larger than 2.0 cm s^−1^ for at least 0.5 s.^[^ ^33^, ^34^ ^]^ Behavioral parameters including trajectory, speed, and total distance were extracted using a commercial software Toxtrac.

### Immunohistochemical Fluorescent Staining

60 min after optogenetic stimulation, mice were perfused with PBS followed by 4% paraformaldehyde (PFA) (cat. no. P1110, Solarbio) in PBS. Brain slices 10 µm thick were dissected in paraffin. Slices were then blocked by using 10% normal goat serum + 0.3% Triton in PBS +5% BSA for 2 h at room temperature. Slices were then placed in primary antibody solution (c‐Fos (9F6) Rabbit mAb, Cell Signaling Technology) diluted (1:500) in blocking buffer at 4 °C for 16–18 h. After washes with PBS, slices were incubated with secondary antibody (Goat anti‐Rabbit IgG (H+L) Cross‐Adsorbed Secondary Antibody, Alexa Fluor 488, Invitrogen) diluted (1:1000) in the blocking buffer for 2 h at room temperature, followed by washes with PBS. Slices were allowed to dry and mounted on glass slides with Prolong Diamond Antifade Mountant with DAPI, capped with a coverslip and then imaged. Samples were imaged by using an upright confocal microscope, in the UBSN facilities of The Hong Kong Polytechnic University. Cells showing both c‐Fos and mCherry signals were counted manually in five FOVs in each brain slice, and the percentage of c‐Fos^+^ cells was counted per slice imaged.

### Quantification and Statistical Analysis

All statistical tests in this study were performed using GraphPad Prism 8 for Windows. Details of the statistical analyses performed for each figure are provided in the figure legends. A *p* value of <0.05 or below was considered statistically significant for all experiments.

## Conflict of Interest

The authors declare no conflict of interest.

## Author Contributions

J.G. and Y.W. contributed equally to this work. J.H.G., Y.Z.H., Y.C.L., and L.S. assisted in conceptualization and design; J.H.G., Y.W., Z.Y.G., F.C., S.K., Z.H.Q., X.Y.Z.H., J.‐J.C.H., D.M.H., T.H.C.H., R.Z., J.J.Z.H., K.F.W., Q.X.X., T.ZH., X.D.H., and Y.C.R. assisted in experiments and/or data analysis; and J.H.G., Y.Z.H., Y.C.L., F.C., S.K., and L.S. assisted in writing—review and editing.

## Supporting information

Supporting Information

## Data Availability

The data that support the findings of this study are available from the corresponding author upon reasonable request.
