# Photonic Nanojet‐Mediated Optogenetics

**DOI:** 10.1002/advs.202204667

**Published:** 2022-09-23

**Authors:** Jinghui Guo, Yong Wu, Zhiyong Gong, Xixi Chen, Fei Cao, Shashwati Kala, Zhihai Qiu, Xinyi Zhao, Jun‐jiang Chen, Dongming He, Taiheng Chen, Rui Zeng, Jiejun Zhu, Kin Fung Wong, Suresh Murugappan, Ting Zhu, Quanxiang Xian, Xuandi Hou, Ye Chun Ruan, Baojun Li, Yu Chao Li, Yao Zhang, Lei Sun

*Adv. Sci*. **2022**, *9*, 2104140

DOI: 10.1002/advs.202104140

In the original published article, there was a mistake in Figure 2I. Please find the correct Figure 2 below.

**Figure 2:**
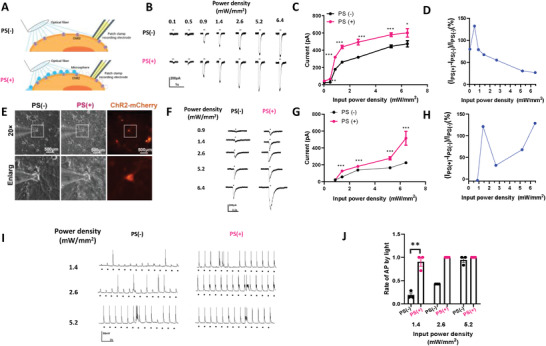
PS microsphere enhances light‐evoked neuron activity via ChR2. A) Schematic configuration for in vitro experiment. Whole‐cell patch clamp recording was conducted in ChR2‐expressed cells stimulated by blue light in the presence of PS (PS(+)) or not (PS(−)). B) Inward currents induced by a 100 ms light pulse with or without PS in 293T cell with ChR2. C) The curve between input power density and inward currents induced by light with PS or without PS in 293T cells, *n* = 5, *p < 0.05, **p < 0.01, ***p < 0.001, unpaired two‐tailed *t*‐test. D) Inward currents of the target neurons as a function of the input power density. E) Primary hippocampal neurons expressing ChR2‐mCherry in bright filed and 488 nm excitation without PS or with PS (scale bar 100 µm). F) Inward currents induced by 20 ms light pulse with or without PS in primary hippocampus neuron with ChR2. *n* = 10, ***p < 0.001 unpaired two‐tailed *t*‐test. G) The curve between input power density and inward currents induced by light with PS or without PS in primary hippocampus neuron. H) Inward currents of the target neurons as a function of the input power density. I) Current clamp recording of hippocampus neuron stimulated with 20 ms pulse light without or with PS. J) Success rate of generation of action potential evoked by light pulse in different power density. *n* = 3, **p < 0.01, unpaired two‐tailed *t*‐test.

The authors apologize for any inconvenience caused.

