# Supplementary material for: Photonic Nanojet‐Mediated Optogenetics
Source: Adv Sci (Weinh). 2022 Feb 20;9(12):2104140. doi: 10.1002/advs.202104140 (PMC9036029; doi:10.1002/advs.202104140)
Supplement: Supplementary file 1 — Supporting Information [file ADVS-9-2104140-s001.pdf]

Supplementary Information

Supplementary Figure 1

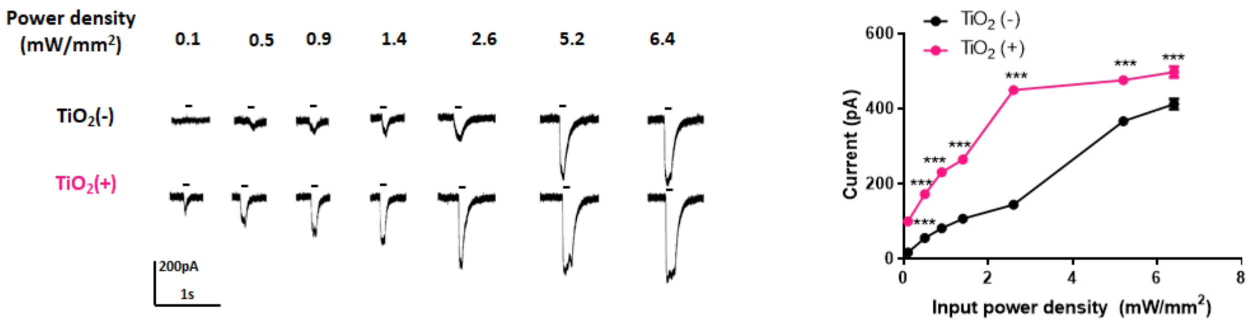

Supplementary Figure 1. Effect of TiO_2 in the whole-cell currents induced by blue light pulse with different power density. (A) Whole-cell current recording in ChR2 transfected 293T cells induced by blue light pulse with different power density with (TiO_2(+)) or without (TiO_2(-)). (B) The curve between input power density and inward currents induced by light with TiO_2 or without TiO_2 in 293T cells, n=9, ***:p<0.001, unpaired two-tailed t test.
